# Current insights on social media as a tool for the dissemination of research and education in surgery: a narrative review

**DOI:** 10.1007/s00595-024-02891-1

**Published:** 2024-07-09

**Authors:** Takehito Yamamoto, Kentaro Goto, Shoichi Kitano, Yurina Maeshima, Toshiyuki Yamada, Yoko Azuma, Shintaro Okumura, Naonori Kawakubo, Eiji Tanaka, Kazutaka Obama, Kojiro Taura, Hiroaki Terajima, Tatsuro Tajiri

**Affiliations:** 1 Public Relations Committee, Japan Surgical Society, Tokyo, Japan; 2 Department of Surgery, Graduate School of Medicine, Kyoto University, Kyoto, Japan; 3 Department of Gastroenterological Surgery and Oncology, Medical Research Institute Kitano Hospital, Osaka, Japan; 4 Department of Breast Surgery, Graduate School of Medicine, Kyoto University, Kyoto, Japan; 5 Department of Cardiovascular Surgery, Nagoya City University Midori Municipal Hospital, Nagoya, Japan; 6 Division of Chest Surgery, Department of Surgery, Toho University School of Medicine, Tokyo, Japan; 7 Department of Pediatric Surgery, Graduate School of Medical Sciences, Kyushu University, Fukuoka, Japan

**Keywords:** Social media, General surgery, Visual abstract, Hashtag, Twitter, Facebook, Instagram, YouTube

## Abstract

The purpose of our narrative review is to summarize the utilization of social media (SoMe) platforms for research communication within the field of surgery. We searched the PubMed database for articles in the last decade that discuss the utilization of SoMe in surgery and then categorized the diverse purposes of SoMe. SoMe proved to be a powerful tool for disseminating articles. Employing strategic methods like visual abstracts enhances article citation rates, the impact factor, h-index, and Altmetric score (an emerging alternative metric that comprehensively and instantly quantifies the social impact of scientific papers). SoMe also proved valuable for surgical education, with online videos shared widely for surgical training. However, it is essential to acknowledge the associated risk of inconsistency in quality. Moreover, SoMe facilitates discussion on specific topics through hashtags or closed groups and is instrumental in recruiting surgeons, with over half of general surgery residency programs in the US efficiently leveraging these platforms to attract the attention of potential candidates. Thus, there is a wealth of evidence supporting the effective use of SoMe for surgeons. In the contemporary era where SoMe is widely utilized, surgeons should be well-versed in this evidence.

## Introduction

Social media (SoMe) has changed communication worldwide. In surgery, SoMe facilitates rapid communication, knowledge sharing among users, the unlimited storage of videos or images in a web-based environment at minimal or no cost, and the borderless connection of surgeons and medical students interested in surgery [1–5]. The importance of information sharing and efficient collaboration with other domains is being increasingly recognized, particularly with the proliferation of rapidly evolving novel technologies such as surgical robots and artificial intelligence. SoMe can be effectively utilized and has attracted attention in this context. For example, the BJS Academy, the educational arm of *the British Journal of Surgery* Society, regularly announces and celebrates, on its webpage, the top 20 SoMe posts each month that garnered the highest number of impressions [6].

**Fig. 1:**
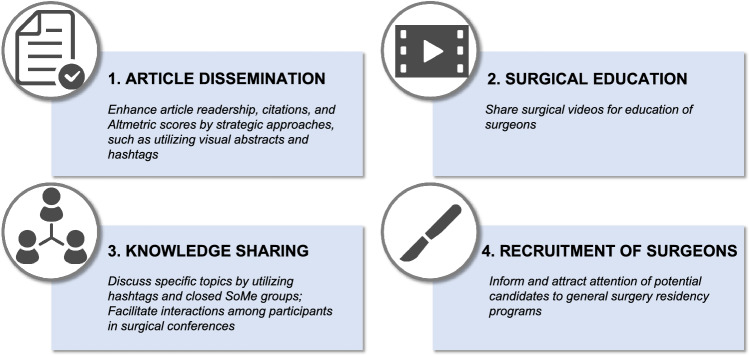
The landscape of academic utilization of social media in surgery

In the present review, we highlight the significance of SoMe in the field of general surgery through the following perspectives (Fig. 1): the dissemination of journal articles, surgical education, the sharing of knowledge and academic debates with other surgeons, and the recruitment of surgeons to the training programs. We also discuss the risks and future perspectives of SoMe, which surgeons must bear in mind.

The first author of this paper (TY), a colorectal surgeon in Japan, has more than 100,000 followers on Twitter (now called “X,” account name: @keiyou30). However, there are some differences between how SoMe is used in Asian countries, including Japan, and how it is used in Western countries. Many people in Asian countries use SoMe in their native language and not English, which often limits the dissemination of information to within their own country. Consequently, while most academic societies and journals in the West maintain their own SoMe accounts in English, this is rare in Asian countries. Moreover, Japanese professionals in the medical field often use SoMe for public education and information dissemination to the general public. As a result, many of their followers on SoMe are citizens. For these reasons, the opportunity for academic information, such as medical papers, to be shared internationally from Japan on SoMe is limited. This review aims to provide information about SoMe to surgeons who are unfamiliar with its academic use in the field of surgery.

## Article dissemination

### Enhancing article citations

Several medical journals have dedicated SoMe accounts on which they regularly post research articles. A previous study found that journals on surgery with dedicated X accounts had a significantly higher impact factor than journals without X accounts and concluded that increased X activity could have helped lift the impact factor [7]. SoMe is an exceptionally effective method of disseminating articles. Some previous articles reported that SoMe exposure increased the number of citations of journal articles [8–10]. For example, Chaus et al. investigated 169 articles in three vascular surgery journals and revealed that Facebook and X’s usage resulted in over a twofold increase in the number of citations [8]. Similarly, in their survey of 404 articles from three coloproctology journals, Jeong et al. reported that articles featured on X had significantly higher citation rates (about 2.8 times) and that X exposure was an independent predictor of a higher citation level (defined as ≥ 5 citations) [9].

The strategic utilization of SoMe is crucial for article dissemination. A retrospective study using data from *Colorectal Disease*, one of the leading journals of coloproctology, showed that a dedicated SoMe strategy improved a journal’s impact, with a 20-fold increase in article downloads, an 11% increase in new subscribers, a 24% increase in article submission, and a 0.9 increase in impact factor between pre- and post-SoMe intervention [11].

Before introducing academic SoMe outreach, an examination of successful cases may prove useful. For instance, Cabrera et al. presented concrete methodologies for surgeons to use X and reported favorable results [12]. Considering the impact of SoMe on the surgical field, the question arises: Should surgeons maintain their own SoMe accounts and share their work on these platforms? Coret et al. addressed this inquiry by evaluating correlations between specific activities on SoMe and the h-index, a metric of scholarly impact, among 722 surgeons in the US and Canada. They found that the number of followers, the number of people followed, and the frequency of posts were independently associated with a higher h-index [13]. Similarly, Elson et al. investigated 88 general surgery SoMe influencers from all over the world, 90% of whom were from the US, Canada, and European countries. They reported that the influencer score, based on several factors, including the number of followers/followings, views, likes, and reposts, was correlated with both the h-index and the number of publications [14]. Surgeons’ active engagement with SoMe may be effective in garnering a wider readership for their research papers and enhancing their standing as surgical scientists.

### Effectiveness of visual abstracts

Visual abstracts are effective for disseminating articles on SoMe. These are graphical summaries that consist of key figures and short texts indicating the background, methods, and outcomes of studies, so readers can grasp the contents of the articles. Since the *Annals of Surgery* introduced visual abstracts in 2016, many journals have adopted these, and now, more than 100 journals and institutions use visual abstracts [15]. Some surgical journals, including the *Journal of American College of Surgeons* and *JAMA Surgery*, require authors to submit visual abstracts, while others, including *Diseases of the Colon & Rectum*, employ in-house creators of visual abstracts.

Considerable evidence exists about the effectiveness of visual abstracts. A prospective case–control study that enrolled 44 original articles in *Annals of Surgery* showed that articles posted with visual abstracts on X gained a 7.7-fold increase in impressions, an 8.4-fold increase in reposts, and a 2.7-fold increase in article visits [16]. Furthermore, a randomized controlled trial (RCT) in *the British Journal of Surgery*, enrolling 41 articles, revealed that visual abstracts had a significantly greater number of total engagements on X; namely, likes, reposts, and replies, as well as impressions and link clicks, than those of plain English abstracts [17]. A recent RCT compared the effectiveness of different types of visual abstracts on X and Facebook, linking visual abstracts, expandable visual abstracts, and article figures. The study enrolled 205 RCT articles with visual abstracts in 12 *JAMA Network* journals [18], and the authors reported that the number of link clicks was significantly higher for linking visual abstracts than for any other type, especially on X.

Although visual abstracts are an important method for conveying research discoveries to readers, their quality varies among journals. Keegan et al. evaluated the accuracy and design of 1325 visual abstracts from 25 surgical journals and proposed a checklist to standardize the quality of visual abstracts to avoid misinformation [19]. Although the influence of visual abstract quality on the dissemination and citation rates of academic papers is undetermined, we considered that conformance to these guidelines may be crucial to effectively disseminate articles when introducing visual abstracts [20].

### Altmetric

Traditionally, scientific papers are evaluated based on the number of times they have been cited. The impact factor of journals and authors’ h-index are widely used metrics based on the citation counts of research papers. Now that diverse information dissemination tools such as SoMe, web news, and blogs are widespread, the impact of papers should be evaluated comprehensively from multiple perspectives.

Altmetric is a new alternative metric that comprehensively and instantly quantifies the impact of scientific papers on society based on several parameters, including the number of page views on the webpage, downloads of the paper’s PDF, citations on SoMe, and mentions in the mass media. Currently, many major journals have introduced Altmetric and a growing trend exists of displaying Altmetric scores prominently on journal webpages. For example, both the *Annals of Surgery* and the *Journal of Clinical Oncology* display the Altmetric score prominantly alongside the article title at the top of their respective pages. Similarly, on the JAMA Network journal webpage, the Altmetric score is featured prominently above the article title, providing readers with immediate insight into the social impact of the research. Although Altmetric is associated with conventional metrics like citation numbers, the h-index, and the impact factor [7, 21–23], many previous articles have proven that it is also strongly correlated to SoMe attention [24–26]. Luc et al. reported the result of an RCT that compared original articles posted on X and those not posted, and stated that posted articles achieved significantly higher Altmetric scores and obtained more citations within 1 year of publication [25]. Luc et al. also investigated 50 articles with the highest Altmetric scores in the *Annals of Thoracic Surgery* in 2013, 2015, and 2017. They revealed that being posted on X or mentioned by news outlets was an independent predictor of high Altmetric scores [24].

Other important findings related to Altmetric scores have been reported. Research papers based on RCTs tend to achieve higher Altmetric scores than other types of papers [27], which means that it may be advantageous to actively disseminate papers related to RCTs through SoMe. Notably, Altmetric scores are independently predictive of being cited by public policy articles, which means that Altmetric can be considered a crucial indicator for assessing the societal impact of medical research papers [28]. Thus, Altmetric offers a significant advantage by providing an immediate quantification of a paper’s social impact; however, it has several important drawbacks. First, it just reflects online attention and does not always indicate the quality of a paper. Second, it tends to favor articles that are popular among the general public, which can lead to a commercial bias. Third, it does not always clearly show the intent or purpose behind the SoMe citations which is a disadvantage compared with traditional academic citations. Understanding these limitations, it is advisable to use Altmetric as just one of the several indicators of impact.

## Surgical education

SoMe is a useful and effective tool for surgical education [29]. This trend has become particularly pronounced in the context of the coronavirus disease 2019 (COVID-19) pandemic, which made face-to-face education challenging [30–32]. In recent years, the widespread adoption of laparoscopic and robotic surgeries has made the review of surgical videos easier. Trainees can enhance their skills through various surgical videos. SoMe also allows for the easy sharing of surgical videos and has become a crucial tool in surgical education.

YouTube, founded in 2015, is a SoMe platform that has become a popular educational tool for storing and sharing surgical videos. According to a previous survey involving surgeons and medical students in the US, 90% used surgical videos to prepare for surgery, with YouTube being the most common platform [33]. With YouTube’s global reach, surgeons can now “see one, do one, teach one, and post one,” revolutionizing surgical education [34].

It should be noted that YouTube content differs from video articles published in academic journals as it lacks formal peer review; therefore, its use requires caution because of the variability in quality [1]. As shown in Table 1, several tools and guidelines for evaluating surgical video quality have been used in previous studies. Reports have reviewed the quality of surgical videos on YouTube, including those on esophagectomy [35], laparoscopic gastrectomy [36], laparoscopic hernia surgery [37–39], sleeve gastrectomy [40], and thoracoscopic sympathectomy [41], and most have posed caution regarding the inconsistency of quality. It was reported that the quality of most YouTube videos on bariatric surgery was reliable and misleading information was not found [42], whereas many popular videos of laparoscopic cholecystectomy were of low quality [43–45]. Notably, junior residents and medical students tended to overvalue the quality of YouTube videos on laparoscopic cholecystectomy [44]. In an investigation of YouTube videos on laparoscopic appendectomy, de Angelis et al. reported greater variability in assessments from trainees than from experienced surgeons [46]. These results suggest that in surgical training using YouTube videos, educators should guide trainees to reliable educational videos with care.

**Table 1:** Common tools and guidelines for evaluating the quality of surgical videos

Instruments	Assessment
*Assessment tools for the information and skills*	
DISCERN [106]	The quality of medical information with 16 items, each scoring 1 to 5
JAMA benchmark criteria [107]	The quality of medical information on the internet from four aspects: authorship, attribution, disclosure, and currency
GOALS (global operative assessment of laparoscopic skills) [108]	The quality of laparoscopic skills with five items, each scoring 1 to 5
LAP-VEGaS (laparoscopic surgery video educational guidelines) [109]	The quality of laparoscopic skills with nine items, each scoring 0 to 2
GQS (global quality score)	The quality of information with a 5-point scale ranging from 1 to 5
OSATS (objective structured assessment of technical skills) [110]	The quality of operative performance with seven items, each scoring 1 to 5
*Assessment tools for the popularity of videos*	
VPI (video power index)	The popularity of videos is calculated by the formula: (like count/dislike count + like count) × 100
*Examples of procedure-specific scales*	
CVS (critical view of safety) score [111]	The quality of laparoscopic cholecystectomy videos with three items
ESS (esophagectomy scoring system) [35]	The quality of esophagectomy videos with 15 items
GOALS-GH (global operative assessment of laparoscopic skills of groin hernia) [112]	The quality of laparoscopic skills for groin hernia with five items
OCRS (objective component rating scale) [113]	The quality of laparoscopic fundoplication with seven items
SGSS (sleeve gastrectomy scoring system) [40]	The quality of sleeve gastrectomy videos with 24 items

Websurg, a widely known educational surgical video platform, was launched in 2000 by IRCAD (Institut de Recherche contre les Cancers de l’Appareil Digestif). IRCAD is a renowned research institute that specializes in minimally invasive surgery research and education. Founded in 1994 in France, it offers advanced training for surgeons worldwide and fosters innovation in surgical technologies through global partnerships and state-of-the-art facilities. The key difference between WebSurg and YouTube is that the former employs a peer-review process, whereas the latter does not. This process is believed to ensure higher quality content than YouTube. Indeed, several studies have demonstrated that videos on WebSurg are more accurate than those on YouTube [47, 48]. Although evidence is limited, WebSurg may be a better option than YouTube for video surgery education.

## Knowledge sharing and academic debate

### Utility of hashtags

Hashtags are useful for participating in discussions on specific topics and aggregating posts related to issues [4]. Table 2 illustrates the creation of numerous hashtags aimed at initiating discussions within the surgical community. The most widespread and successful hashtag is #SoMe4Surgery, which was created in 2018 and used in over 10,000 posts and 30,000 reposts in the first 5 months [49]. Using this hashtag, surgeons from all over the world have conversations with each other on diverse surgical topics, mentorship, collaboration in clinical research, development of skills in drafting manuscripts, contents of medical journals, and more [50]. The X account of SoMe4Surgery (@me4_so) was created in 2018 and has over 10,000 followers, fostering discussions related to surgical research, practice, and education. Another successful hashtag is #TSSMN, which was created in 2015 by The Thoracic Surgery Social Media Network (TSSMN) with the aim of garnering attention for articles from *The Annals of Thoracic Surgery* and *The Journal of Thoracic and Cardiovascular Surgery*, which are prominent journals in cardiothoracic surgery. SoMe users can stay updated on the latest information in this field by following the hashtag #TSSMN and X account (@TSSMN) [51, 52]. The TSSMN conducted a successful TweetChat for a journal club using this hashtag [53]. A Tweetchat is a scheduled X discussion in which a selected paper is announced to followers beforehand, and the authors themselves participate in the discussion. In a previous TweetChat, which focused on supporting cardiothoracic surgeons during the COVID-19 pandemic, 273 participants joined the discussion, and their impressions reached 7.5 million people [54].

**Table 2:** Useful and active social media hashtags in surgery

Hashtags	Aim	Created in
#SoMe4Surgery	To discuss diverse topics in surgery	2018
#colorectalsurgery	To activate the global community of colorectal surgeons and collate discussions on coloproctology	2016
#colorectalresearch	To promote articles from four major journals (*Diseases of the Colon & Rectum*, *Colorectal Disease*, *the British Journal of Surgery*, and *Techniques in Coloproctology*)	2016
#TSSMN	To promote articles from *The Annals of Thoracic Surgery* and *The Journal of Thoracic and Cardiovascular Surgery*	2015
#globalsurgery	To discuss diverse topics in surgery	2010
#surgicallresearch	To discuss diverse topics in surgery	2013
#SoMeHPB	To discuss diverse topics in hepatopancreatobiliary surgery	2019
#visualabstract	To enhance the rationale of the visual abstracts	2016
#Donatelife	To promote the dissemination of transplant medicine	2012
#IlookLikeASurgeon	To discuss the gender diversity in surgery	2015

The hashtag #colorectalsurgery has a great impact on colorectal surgeons through SoMe. It was created in 2016 with the aim of activating the global community of colorectal surgeons and collating discussions on coloproctology [55]. This hashtag attracted significant attention during major international colorectal surgical conferences, and related posts reached over 60 million impressions in the first 180 days [55]. However, owing to the widespread adoption of this hashtag, an excess of unrelated noise has rendered the accurate selection of information for research on colorectal surgery difficult. Consequently, a new hashtag, #colorectalresearch, was introduced with a specific focus on colorectal surgery, aiming to promote articles from four major journals (*Diseases of the Colon & Rectum*, *Colorectal Disease*, *the British Journal of Surgery*, and *Techniques in Coloproctology*) [56]. This incident highlights the risk of information overload on SoMe, increasing the difficulty of discerning relevant information [57].

Navarro et al. investigated 2633 posts with the hashtag #GlobalSurgery and revealed a significant finding that the top 20% of X and Instagram influencers were responsible for 67% of posts, respectively [58]. This suggests that the viewpoints expressed by a minority of users, prominently visible on SoMe, may not reflect a diverse array of perspectives. This underscores another risk associated with hashtags that users should be mindful of. Numerous other active hashtags on SoMe cater to specific topics in surgery. For instance, #SoMe4HPB was established in 2019 to center discussions on hepatopancreatobiliary surgery [59], #visualabstract was created by *Annals of Surgery* to enhance the rationale of visual abstracts, #IlookLikeASurgeon was created to discuss gender diversity in surgery [60], and #donatelife was created in 2012 with the X account @Donatelife (currently having over 14,000 followers) to promote the dissemination of transplant medicine [61].

### Academic debates in closed Facebook groups

SoMe is a useful tool used by surgeons to exchange surgical knowledge on particular diseases and surgical procedures. Facebook is considered the most suitable platform for closed communication among surgeons. In a prior survey examining Facebook groups tailored for medical students, general surgery residents, and practicing general surgeons, findings revealed that 59.5% of participants shared challenging cases for discussion with their peers, while 78.6% offered advice and insights by posting comments [62].

In 2012, the International Hernia Collaboration (IHC) became the first Facebook group created for discussions on hernia management. It now has more than 13,000 members seeking advice for difficult hernia cases. Bernardi et al. investigated 598 unique responses to 60 clinical threads using the IHC and reported that 96.6% of the responses were safe [63]. The network of surgeons within the IHC also serves as a valuable resource for referring patients to specialized facilities within their own country or even other countries for advanced care [64].

Since the development of the IHC, several other closed Facebook groups involving many surgeons have been created. For example, in 2015, a Facebook group for the Online Society of American Gastrointestinal and Endoscopic Surgeons (SAGES) called the SAGES Foregut Surgery Masters Program focused on discussing surgery of the esophagus, stomach, and small intestine. Surgeons from 40 countries participate in this group, with the most discussed topic being operative strategy, followed by patient management [65]. SAGES also manages several Facebook groups for various surgical specialties, such as colorectal, bariatric, hepatobiliary pancreatic, and acute care surgery. These groups attract approximately 2,000–5,000 members each.

The COVID-19 pandemic intensified surgeons’ engagement in Facebook groups. Docimo et al. reported a significant (5–13%) increase in memberships in the eight SAGES closed Facebook groups from before the pandemic [31]. In 2015, a Facebook group called the “Robotic Surgery Collaboration” was created for general surgeons using robotics and the robotic industry to improve patient outcomes. It now has more than 15,000 members. Myers et al. investigated the use and engagement patterns of this group and revealed that the number of posts per day was greater on midweek days than on other days [66].

In online academic information exchange, it is preferable to make comments using real names from the perspectives of reliability and ethics. In fact, almost all physicians participating in Facebook groups disclose their real names and affiliations, and most interactions on X are also conducted using real names. Members must maintain professionalism and patient confidentiality. The SAGES Facebook Group Task Force endorses the professional use of the closed SoMe group platform and recommends the use of a public disclaimer and an informed consent template [67].

### Discussion in conferences

Although the use of SoMe at scientific conferences can facilitate instant interaction among physicians and the dissemination of findings, few reports on this topic in surgery exist [68]. In other fields, SoMe influencers have played important roles in the promotion of discussion during conferences in anesthesiology [69], urology [70], and cardiology [71]. Nolte et al. reported that research presented at conferences of the American Urological Association with more SoMe engagements were significantly more likely to be published [70]. However, sharing conference slides on SoMe requires careful consideration of intellectual property rights. Although a survey targeting hernia surgeons on SoMe revealed that many users were tolerant of sharing presenters’ slides, obtaining the consent of the presenter is recommended when using SoMe at conferences [72].

### Recruitment to clinical trials

The potential benefit of SoMe in the recruitment of patients to surgical clinical trials and enhancement of researcher collaboration has also been reported [73–77]. The COVID-Surg Collaborative Group (X account: @CovidSurg) is the most successful example of this. In a large surgical trial assessing the impact of COVID-19 on surgical outcomes, SoMe played an important role in the recruitment of participants globally in a very short time [76]. This group has more than 8500 X followers, with 1677 centers from 122 countries enrolled and has led to published papers in *The Lancet* [76], the *Journal of Clinical Oncology* [78], and the *British Journal of Surgery* [79–82].

Although the optimal use of SoMe in the promotion of clinical trials still lacks concrete evidence, some studies have indicated that the utilization of specific hashtags, eye-catching logos, and color schemes could increase SoMe engagement and attract attention to clinical trials [74, 75]. Further research on this topic should be conducted.

## Recruitment of surgeons

SoMe platforms are a significant tool for the promotion and engagement of residency programs. In a survey of 613 general surgery residency applicants in the US from 2020 to 2021, 70.9% of respondents indicated that SoMe was a crucial source for obtaining information, and 62.6% indicated that SoMe contributed to cultivating a positive image of training programs [83].

According to previous studies, 47–59% of the general surgery residency programs in the US have SoMe accounts [84–86]. Instagram is the most frequently used platform, followed by X [84]. A total of 40.2% of their program directors and assistant/associate program directors had at least one account on X and/or LinkedIn [85]. This trend was accelerated during the COVID-19 pandemic [86, 87]. Fang et al. investigated 319 general surgery residency programs in the US and found that 188 (59%) had SoMe accounts, 112 (32%) of which were created after the pandemic [86].

Although the majority of posts (70–81%) on general surgery residency programs were promotional, the educational content gained significantly higher engagement [88, 89]. These results suggest that excessive reliance on promotional posts for recruiting surgeons may reduce the effectiveness of dissemination efforts. Hence, maintaining a balance between promotional content and educational materials is imperative for maximizing impact.

Since the SoMe platforms utilized by applicants may vary by age, country, or specialty, a thorough understanding of SoMe usage patterns among young physicians and medical students who are targets for recruitment is essential. For instance, previous reports indicate that most young general surgery trainees in the US, with an average age of 30 years, utilize SoMe platforms such as Instagram, YouTube, and Facebook, with usage rates exceeding 80% across all platforms [90]. In comparison, most of the young general and bariatric surgeons (≤ 45 years) in Mexico prefer Facebook, followed by X and Instagram [91]. There are many other surveys on SoMe usage patterns enrolling surgeons with various specialties, such as visceral [92], colorectal [93–95], hernia [96], and transplantation surgery [97]. In contrast, based on a survey of 110 general surgery program directors in the US, Langenfeld et al. reported that directors frequently viewed the SoMe profiles of students and residents and leveraged their online behavior for ranking purposes during the hiring process [98]. Therefore, the education of young physicians in terms of SoMe professionalism is essential.

## Risks and guidelines

Surgeons who use SoMe must consider its risks and limitations. First, SoMe lacks a peer-review process, and there is no obligation to disclose reliable information or references, which potentially poses a risk of spreading information with insufficient scientific evidence, leading to misunderstandings among non-specialized users [99]. Furthermore, the absence of a mandated disclosure of conflicts of interest may contribute to biased information sharing [99]. Second, SoMe posts may breach confidentiality obligations or be undesirable from the perspective of patient privacy protection. Digne-Malcolm et al. investigated 66 images posted on X in 16 countries and found that half were at risk of breaching confidentiality, with patients being identifiable, and 18 posts including identifiable location information [100]. Another study from Brazil, which included attending surgeons, residents, and graduates, showed that more than 60% of the study participants were unaware of the confidentiality preservation policy [101]. The American College of Surgeons and the Cardiothoracic Ethics Forum provide guidelines for surgeons with respect to confidentiality, disclosure of conflicts of interest, and promotion of professionalism [102–104]. Surgeons who use SoMe must conform to these guidelines.

SoMe influencers with many followers and significant speaking influence may have a considerable impact, even when their academic achievements or contributions are relatively modest [2]. The Kardashian index (K-index) was used to quantify this discrepancy. Neil Hall, the creator of this index, wrote, “If your K-index gets above 5, then it’s time to get off Twitter and write those papers” [105]. When gathering information on SoMe, readers must not be swayed solely by the influence of prominent figures on SoMe, but must diligently verify expertise and information sources.

## Future perspectives

Numerous changes in SoMe have occurred, which surgeons should be aware of. In July, 2023, Twitter underwent rebranding, adopting the name X, and concurrently made ongoing modifications to its algorithm, influencing post impressions on the timeline. In addition, a subscription model was introduced, wherein subscribers paying an annual fee to X enjoy certain advantages, such as the ability to post text exceeding 280 letters. Notably, several academic accounts, including *the British Journal of Surgery,* the American College of Surgeons*,* and Harvard Health, have been registered as paying accounts. Thus, research exploring the optimal management of paying accounts in this new scenario is anticipated.

In August 2023, Google announced that YouTube would remove false information on cancer treatment based on its medical misinformation policy. While this proactive measure may enhance the reliability of YouTube video content, it may also pose a challenge for surgeons who wish to display their videos on the platform.

The new service Threads, released by Meta in July 2023, is a short-text SoMe platform that can leverage existing Instagram accounts. Notably, a substantial number of Threads followers were gained quickly by prestigious English journals such as the *New England Journal of Medicine* with over 86,000 followers, *Nature* with over 98,000 followers, and *JAMA Network* with over 24,000 as of the time of writing this article. Regarding how surgeons should engage in research dissemination and communication, waiting for the establishment of evidence may be prudent in this evolving landscape.

The Japan Surgical Society launched its official Facebook and X accounts in February, 2024, utilizing these platforms to introduce its official journals, promote its annual meeting, and announce seminars for members. Moving forward, the society aims to devise more efficient means to promote events that appeal to young surgeons and medical students on SoMe. Moreover, the society plans to establish a separate SoMe account dedicated to disseminating articles from its official English-language journal, *Surgery Today*, aiming to further enhance its recognition and increase citation numbers.

## Conclusions

SoMe is beneficial for disseminating research findings and fostering connections among surgeons, and is effective for the education and recruitment of surgeons and medical students. While being mindful of certain risks and recent changes, the effective utilization of SoMe is becoming increasingly essential for surgeons.
